# Optical Second Harmonic Generation of Low-Dimensional Semiconductor Materials

**DOI:** 10.3390/nano14080662

**Published:** 2024-04-11

**Authors:** Yue Fu, Zhengyan Liu, Song Yue, Kunpeng Zhang, Ran Wang, Zichen Zhang

**Affiliations:** 1 Microelectronics Instruments and Equipment R&D Center, Institute of Microelectronics, Chinese Academy of Sciences, 3 Beitucheng West Road, Beijing 100029, China; fuyue@ime.ac.cn (Y.F.); liuzhengyan@ime.ac.cn (Z.L.); yuesong@ime.ac.cn (S.Y.); zhangkunpeng@ime.ac.cn (K.Z.); 2 School of Integrated Circuits, University of Chinese Academy of Sciences, No. 19(A) Yuquan Road, Beijing 100049, China

**Keywords:** SHG, low-dimensional materials, time-resolved SHG, perovskite, nanophotonics

## Abstract

In recent years, the phenomenon of optical second harmonic generation (SHG) has attracted significant attention as a pivotal nonlinear optical effect in research. Notably, in low-dimensional materials (LDMs), SHG detection has become an instrumental tool for elucidating nonlinear optical properties due to their pronounced second-order susceptibility and distinct electronic structure. This review offers an exhaustive overview of the generation process and experimental configurations for SHG in such materials. It underscores the latest advancements in harnessing SHG as a sensitive probe for investigating the nonlinear optical attributes of these materials, with a particular focus on its pivotal role in unveiling electronic structures, bandgap characteristics, and crystal symmetry. By analyzing SHG signals, researchers can glean invaluable insights into the microscopic properties of these materials. Furthermore, this paper delves into the applications of optical SHG in imaging and time-resolved experiments. Finally, future directions and challenges toward the improvement in the NLO in LDMs are discussed to provide an outlook in this rapidly developing field, offering crucial perspectives for the design and optimization of pertinent devices.

## 1. Introduction

Franken et al. first researched second harmonic generation (SHG) from crystalline quartz in 1961, marking the inception of nonlinear optical (NLO) properties [1]. SHG represents the fundamental nonlinear course wherein an incident wave with frequency *ω* comes into contact with one nonlinear material, resulting in an emergent wave of double frequency 2*ω* [2]. This distinctive phenomenon has been observed in noncentrosymmetric media under intense light fields [3,4,5,6] and garnered significant interest in photonic and optoelectronic device applications [7,8], materials characterization [9,10], and optical frequency converters [11,12]. The Kurtz–Perry powder technique can evaluate second harmonic generation (SHG) intensity in pristine powder form, saving a significant amount of time and energy in the preliminary screening of materials [13]. Notably, the commercially successful bulk crystals such as Beta Barium Borate (BBO) and Lithium Triborate (LiB_3_O_5_), etc., can be attributed to the exploitation of SHG. However, these conventional materials are ill-suited for the emerging technical requirement of current and future nonlinear optics, especially on-chip nanophotonics. To facilitate chip-scale nonlinear optics, there is a pressing need for nanoscale materials that exhibit robust nonlinear optical responses. Consequently, there is an immediate need for novel materials that offer large nonlinear responses in compact sizes at a reasonable cost that are tailored for multifunctional and high-performance applications in nonlinearity. The field is currently experiencing a substantial shift due to the identification and advancement of low-dimensional materials (LDMs).

To date, significant efforts have been invested in enhancing the linear optical properties of LDMs. However, research into their NLO effects remains nascent. The exploration of NLO effects at the low-dimensional (LD) scale has provided a fresh perspective on the study of LDM optical properties. A fundamental nonlinear optical effect, integral to a myriad of applications, is the second-order NLO process referred to as SHG. Recently, SHG has been identified in numerous two-dimensional (2D) layered materials. This phenomenon is attributed to the intrinsic noncentrosymmetric structures of these materials, which exhibit SHG effects, for example, 3R MoS_2_ [14], CuInP_2_S_6_, and α-In_2_Se_3_. While some centrosymmetric 2D layered materials (e.g., 2H MoS_2_, 2H WS_2_, and graphene) are expected to have no SHG signal because optical susceptibility tensors vanish, this phenomenon can be observed when certain techniques are employed to transition from centrosymmetric structures to noncentrosymmetric ones. These techniques include adjusting the number of layers [10], introducing an external electric field [15], incorporating and tuning defects [16], as well as artificially stacking heterostructures and homostructures. Furthermore, spontaneous polarization has led to the reported generation of SHG in MAPbI_3_ single-crystal [17] and hybrid germanium iodide perovskite [18], highlighting their potential applications for second-order NLO. The dimension reduction endows LD perovskites with unique band structures compared with their 3D counterparts, which is pivotal in determining their SHG properties [19,20,21]. Researchers have concurrently been engaged in the development of diverse nanomaterials exhibiting high second-order NLO properties, aiming to apply them in chemical and biological detection, as well as in photonics [22,23,24,25]. Additionally, it has been observed that the second-order nonlinear susceptibility of atomically LD materials aligns with that of conventional bulk NLO materials. This alignment suggests potential for innovative applications in optoelectronic and photonic devices. The unique combination of unparalleled material compatibility and seamless integration, along with their varied optoelectronic properties, positions LD SHG materials as viable candidates for nanophotonic devices incorporated into forthcoming chips.

**Figure 1:**
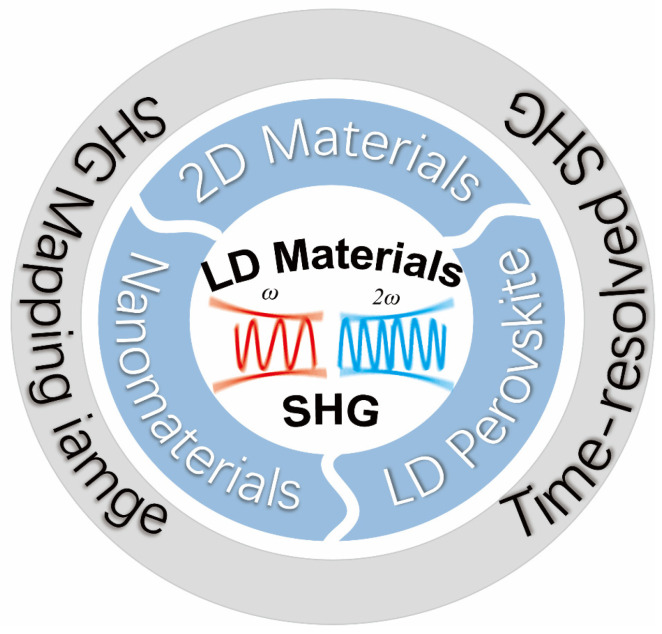
Summary of SHG in 2D materials, LD perovskites, and nanomaterials and their corresponding applications of SHG in LD materials.

Several reviews have been conducted on the nonlinear optical properties of LDMs [3,4,26,27], with a few recent papers offering in-depth discussions specifically on the SHG in 2D materials [28,29,30]. In this paper, we provide a thorough review of recent advancements in the investigation of highly efficient and adjustable SHG processes in LDMs. In Section 2, the fundamental theory bases of SHG for LDMs and the experimental method for second-order nonlinear LDMs are succinctly introduced. In Section 3, the modulation and enhancement of SHG response strategies and their advances in LDMs, including 2DLMs, LD perovskites, and nanomaterials, are reviewed systematically. In Section 4, we present the development of more multifunctional and practical LD materials in optical characterizations and applications. In Section 5, the current state and challenges of SHG for LD materials as well as practical applications of LDMs in nonlinear integrated devices are discussed. In this review, we offer a comprehensive overview of the topics discussed as shown in Figure 1.

## 2. Theoretical Background

### 2.1. Basic Concept of SHG

Nonlinear optical processes can be understood by expanding the polarization $P\left(r,t\right)$ of the material in terms of a Taylor series relative to the incident light field $E\left(r,t\right)\;$ [31].

$$P\left(r,t\right)=P_{L}\left(r,t\right)+P_{NL}\left(r,t\right)$$

Here $P_{L}\left(r,t\right)$ is the linear polarization component. When weak light propagates through the material, the nonlinear polarization component can be neglected, and only linear optical processes are considered.

$$P_{L}\left(r,t\right)=\chi^{\left(1\right)}E\left(r,t\right)$$

$\chi^{\left(1\right)}$ represents the material’s linear susceptibility, reflecting the physical processes of the linear optical response of the medium to light (such as linear refraction and linear absorption). Its real part represents the linear refractive index, and the imaginary part represents the linear absorption coefficient.

$P_{NL}\left(r,t\right)$ is the nonlinear polarization component. When intense light propagates through the medium (typically with $E\left(r,t\right)$ in the range of $10^{15}-10^{18}\;V/m$), nonlinear optical processes occur [31].

(1) $$P_{NL}\left(r,t\right)=P^{2}\left(r,t\right)+P^{3}\left(r,t\right)+\ldots +P^{n}\left(r,t\right)=\chi^{\left(2\right)}E{\left(r,t\right)}^{2}+\chi^{\left(3\right)}E{\left(r,t\right)}^{3}+\ldots +\chi^{\left(n\right)}E{\left(r,t\right)}^{n}$$

$\chi^{\left(2\right)},\chi^{\left(3\right)}$ represent the second-order, and third-order nonlinear polarization susceptibilities of the medium, respectively. Here, n signifies an integer value.

The relationship between second-order polarization and second-order response is as follows:

(2) $$P^{2}\left(r,t\right)=\chi^{\left(2\right)}E{\left(r,t\right)}^{2}$$

$\chi^{\left(2\right)}$ reflects second-order nonlinear optical effects, such as difference frequency generation, second-harmonic generation [29], and so on.

**Figure 2:**
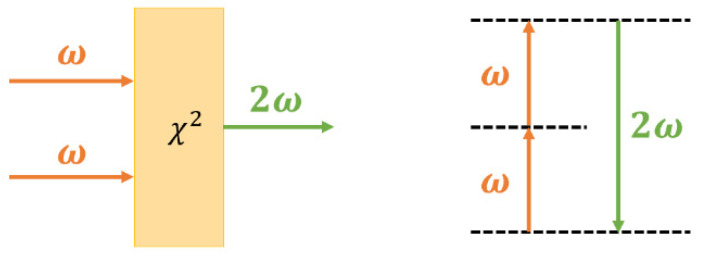
Diagrammatic representation of the second harmonic generation principle.

From a polarization perspective, it can be understood that the incident light with a frequency of *ω* interacts with the second-order polarization susceptibility of the material, inducing polarization and resulting in a second harmonic light beam with a frequency of 2*ω* [32,33,34,35]. The process of SHG can also be elucidated through the interaction of atomic energy levels and photons: electrons absorb two photons with the same frequency, transition to a higher energy level, and then emit a photon with a frequency of 2*ω* when transitioning back down. This phenomenon is the second harmonic signal (Figure 2).

During spatial inversion symmetry operations, the vectors change the positive and negative sign in both the second harmonic intensity and the fundamental frequency incident light intensity.

(3) $$P^{2}\left(E\right)={-P}^{2}\left(-E\right)$$

(4) $$\chi^{\left(2\right)}EE=-\chi^{\left(2\right)}(-E)(-E)$$

(5) $$\chi^{\left(2\right)}EE=-\chi^{\left(2\right)}EE$$

Therefore, the calculated polarization intensity remains unchanged, leading $\;\chi^{\left(2\right)}$ = 0. That is, for materials with centrosymmetric symmetry, their second-order polarization is zero, and thus no SHG signal is generated [36]. If a material generates the SHG signal, it indicates that the centrosymmetric symmetry of the material is broken, such as at interfaces and defects. Thus, the structural symmetry of a material can be characterized through the phenomenon of second harmonic generation.

The SHG and THG signals generated by the crystal under intensive laser are also affected by the crystal structures of the ultra-thin flakes. Additionally, for low-dimensional materials, the linear as well as the nonlinear optical properties near the band edge are dominated by the exciton effect. When the exciton states are involved as intermediate or final states for the nonlinear transitions, the NLO can be enhanced dramatically. Therefore, the enhanced excitonic effect in low-dimensional systems also strongly influences the harmonic generation intensity [37,38,39]. Previous studies of 2D materials reveal that, when the two-photon excitation energy is in resonance with the exciton energy, the SHG efficiency is shown to increase up to three orders compared with the excitation off-resonance [37,38].

The frequency conversion efficiency of semiconductor materials’ nonlinear optical processes exhibits a high degree of dispersion. This is because the material’s nonlinear susceptibility varies with the incident light frequency [40,41]. Additionally, the utilization of resonance effects can significantly enhance the interaction between light and matter in two-dimensional materials, greatly improving the efficiency of second harmonic generation [37,42,43]. Particularly, when the incident light frequency matches the resonance frequency of the material’s electrons or excitons, the frequency conversion is most effective, as electron transitions occur between real electronic states. Therefore, the response of the second-order nonlinear susceptibility is dispersive, influenced by factors such as the distribution of electronic states, bandgap structure, and lattice symmetry.

Despite the intense experimental studies of the SHG effects in Mie-resonant nanostructures, a comprehensive theory of the SHG emission from nanoparticles with nonzero bulk nonlinearity tensor $\chi^{\left(2\right)}$ has not been proposed yet [44]. Resonant nanoparticles can enhance the electric field, thus improving the generation efficiency of nonlinear signals. This is because, at the nanoscale, the nonlinearity of light is often associated with geometric plasma resonances in plasma nanostructures. At the same time, the directivity of the second harmonic emission can be controlled. Under the resonance excitation of a single magnetic dipole mode, the directivity of the second harmonic emission can be controlled by rotating the dipole moment relative to the material crystal lattice. The mechanisms in resonant enhancement and SHG of nanoparticles are dependent on the symmetry of the crystalline structure and polarization of the incident light.

### 2.2. Experiment Method

Second harmonic generation (SHG) provides a non-invasive, straightforward, and efficient characterization method for determining the properties of low-dimensional materials. It can be employed to identify characteristics [45,46,47,48,49,50,51,52,53,54,55], such as the layer number, crystal lattice symmetry and orientation, twist angle, strain direction, and intensity.

**Figure 3:**
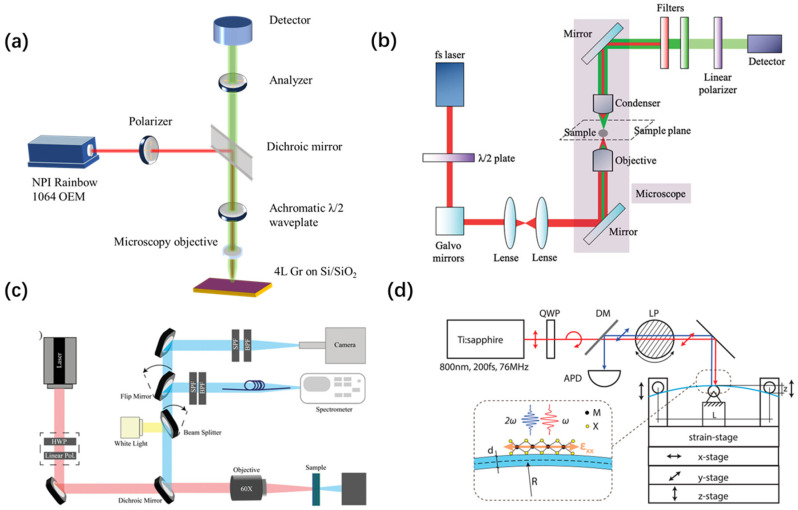
Experimental setups. (**a**) Schematic diagram illustrating the fundamental optical setup for reflective second harmonic generation (SHG) measurements [54]. (**b**) Schematic diagram illustrating the fundamental optical setup for transmissive second harmonic generation (SHG) measurements [56]. Copyright 2022, Wiley-VCH GmbH. (**c**) The optical spectroscopy setup is utilized to gather both intensity maps and spectral data [60]. Copyright 2023, the Authors. Advanced Optical Materials published by Wiley-VCH GmbH. (**d**) Schematic diagram of the optical setup for measuring strain effect on SHG [61]. Copyright 2018, the Authors. This article is distributed under a Creative Commons Attribution (CC BY) license.

The fundamental method for SHG measurement is illustrated in Figure 3a [54]. A laser generates laser beams with a specific excitation wavelength, which are polarized after passing through a polarizer to achieve a specific linear polarization state. Subsequently, a dichroic mirror is used to selectively ensure that the laser beams of that wavelength only undergo reflection and not transmission. The reflected laser beams, after passing through a birefringent half-wave plate for rotation of linear polarization states and phase modulation, are focused onto the sample surface by a microscope objective. During the interaction between the sample and laser, the SHG signal is generated. The microscope objective is utilized to effectively collect these generated SHG signals. Following this, the SHG signals undergo further adjustment of the polarization state through a birefringent half-wave plate. They then pass through a dichroic mirror to ensure exclusive transmission in the optical path. Subsequently, a polarizer selectively allows the light with specific polarization directions to pass through, employing a spectrometer for polarization analysis. Finally, the signals, having passed through the spectrometer, are transmitted to the detector for precise measurement of the SHG signals [49,50,56,57]. In addition, transmission measurements are a common approach in the study of SHG (Figure 3b) [8,45,58,59].

Researchers have initiated improvements in the experimental setup of SHG measurement. Building upon the foundation of Figure 3a, Figure 3c introduces a white light beam to enable the observation of the sample’s morphology, and two detection arms are utilized to acquire intensity maps and spectra of second harmonic generation (SHG) [60]. In Figure 3d, a quarter-wave plate (QWP) is employed to convert linearly polarized light from the laser into circularly polarized light, allowing for polarization state adjustment solely by manipulating the linear polarizer. Additionally, a strain device is positioned on the sample stage to facilitate an in-depth exploration of the impact of stress on SHG signals [61].

## 3. State of the Art of LDM-Based SHG

In low-dimensional systems, strong quantum confinement leads to band gap expansion and thus larger band gaps, which, in turn, affect the third-order NLO strength. The confinement of electrons in low-dimensional systems enhances the interaction between light and matter, resulting in more pronounced linear and nonlinear optical responses than those of 3D bulk materials. Unlike third-order NLO, second-order processes such as SHG necessitate a stringent crystal structure of noncentrosymmetric. Consequently, second-order NLO is frequently associated with ferroelectricity, pyroelectricity, and the Rashba effect. Recently renewed interest in nanoscale SHG is demonstrated in the increasing demand for subwavelength coherent light sources achieved by disrupting symmetry through interfaces [62], imperfect spheres [62], and asymmetric shapes [63]. Furthermore, nanoscale SHG can also be realized through the utilization of noncentrosymmetric nanocrystals [64,65,66,67,68], asymmetric geometric configurations [69], and the application of NLO material coating on nanospheres [70].

### 3.1. 2D Materials

#### 3.1.1. SHG in Graphene

Since the discovery of graphene by Geim et al. [71] in 2004, two-dimensional materials have been at the forefront of research. Graphene refers to an atomic-level thin layer of carbon atoms densely arranged in a hexagonal lattice, serving as the fundamental building block for all other dimensions of graphite materials [72]. Due to its high mechanical strength [73] and excellent electrical [74] and optical properties [75], graphene has emerged as a competitive candidate material for constructing sensors, flexible devices, solar cells, and more [76,77,78]. The monolayer of graphene exhibits a central symmetric structure, and according to the dipole approximation, no second harmonic generation (SHG) response is expected [79]. In response to this, researchers have endeavored to break the inversion symmetry through various methods to induce SHG responses and thereby evoke novel optical properties (Table 1).

**Figure 4:**
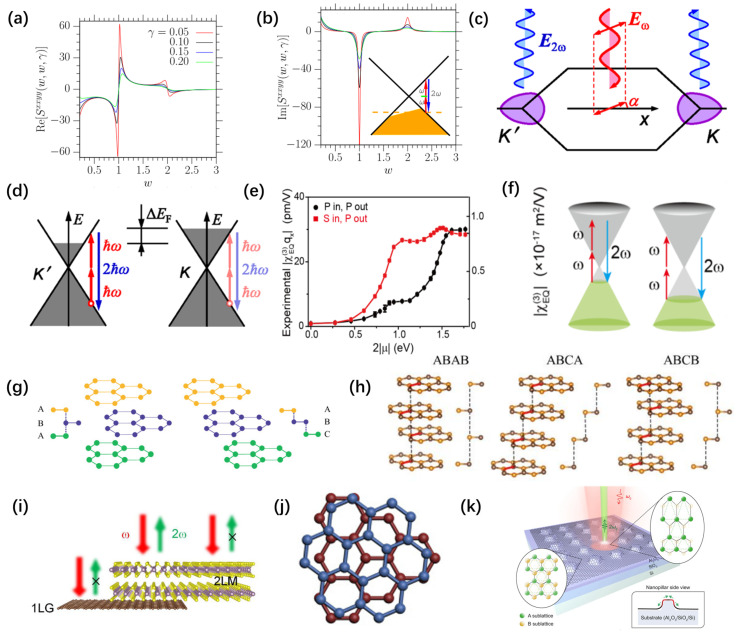
DC current, valley polarization, doping, stacking, twisting, and non-uniformly straining induced SHG in graphene. (**a**) The dependence of Sxxyy (*w*,*w*,*γ*) [(**a**) real and (**b**) imaginary] on the “*ω*” for different *γ* [80]. Copyright 2014, Optical Society of America (Washington, DC, USA). (**c**) SHG arises due to trigonal valley asymmetry, with signals from the Κ and Κ’ valleys exhibiting opposite signs [86]. The red curve represents the incident fundamental frequency, and the blue curves represent the frequency doubled signals. (**d**) Valley population imbalance results in a net SHG signal [86]. Copyright 2014, American Physical Society (College Park, MD, USA). The red line represents the incident fundamental frequency, and the blue line represents the frequency doubled signal. (**e**) The relationship between chemical potential and the effective second-order nonlinear susceptibility of SHG [79]. (**f**) Transition diagrams for Fermi-edge resonances involving one and two-photon processes, respectively [79]. The red line represents the incident fundamental frequency, and the blue line represents the frequency doubled signal. Copyright 2019, American Physical Society. (**g**) Various stacking arrangements of the graphene trilayer, including ABA (Bernal) and ABC (rhombohedral) arrangements [83]. Copyright © 2018, the Authors, some rights reserved; exclusive licensee American Association for the Advancement of Science. (**h**) Various stacking arrangements of graphene tetra-layers, including ABAB, ABCA, and ABCB [54]. (**i**) Heterostructures formed by the van der Waals (vdW) stacking of center-symmetric monolayer graphene and bilayer molybdenum disulfide (vdWH) induced SHG [51]. The red arrow represents the incident fundamental frequency, and the green arrow represents the frequency doubled signal. Copyright © 2023, the Authors, some rights reserved; exclusive licensee American Association for the Advancement of Science. (**j**) Twisted bilayer graphene with broken inversion symmetry [84]. Copyright 2020, Elsevier Inc. (Amsterdam, The Netherlands) (**k**) Non-uniform strain in monolayer graphene-induced SHG [55]. Copyright 2023, the Authors.

For instance, external stimuli such as current and electric fields [80,81,85] can induce SHG responses in graphene. In 2004, Chang et al. [80] induced SHG through direct current, utilizing semiconductor Bloch equations to calculate the displacement of carrier distribution in the Brillouin zone caused by the direct current field. They provided an analytical expression for the nonlinear susceptibility (Figure 4a,b). Similarly, applying an electric field perpendicular to the graphene plane disrupts the sublattice symmetry, leading to the generation of second harmonic waves [85]. Researchers have also observed an inversion relationship between the K and K’ valleys. Under normal incidence plane electromagnetic wave excitation, the second harmonic signals from different valleys have opposite phases. When the valley carrier distribution is uniform, the second harmonic signals cancel out. However, non-uniform carrier distribution results in the generation of second harmonic signals (Figure 4c,d) [86].

Furthermore, doping can induce a transition in graphene’s electronic structure from electric dipole forbidden to electric quadrupole allowed, thereby leading to intense SHG in graphene [79,82]. This novel SHG exhibits properties of dipole response, which is attributed to the effective inversion symmetry breaking caused by the optical coupling of photons within the plane. Zhang et al. [82] reported that charge doping controlled by oxidation–reduction effectively breaks the central symmetry of bilayer graphene (BLG), leading to the generation of strong SHG (Figure 4e,f).

Different stacking configurations can also induce SHG. Shan et al. [83] investigated the three-layer graphene stacking configurations ABA (Bernard) and ABC (rhombus) (Figure 4g). In ABA trilayer graphene, the carbon atoms in the second layer are positioned directly above the centers of the hexagons in the first layer, and the third layer is precisely positioned above the first layer. In contrast, in ABC trilayer graphene, each layer of carbon atoms is offset from the layer below by a certain distance. Therefore, ABA trilayer graphene belongs to the D_3h_ point group, exhibiting inversion symmetry breaking, which allows the observation of SHG signals. On the other hand, ABC trilayer graphene retains a centrosymmetric structure similar to monolayer graphene and does not generate second harmonic signals. Subsequently, Zhou et al. [54] documented the examination of significant nonlinear optical SHG in four-layer graphene stacking with ABCB configuration, while no SHG was observed in isomers with ABAB and ABCA stacking (Figure 4h). Additionally, researchers reported that center-symmetric two-dimensional materials, for example, van der Waals (vdW) stacking of bilayer molybdenum disulfide (2 LM) and monolayer graphene (1 LG), can promote substantial SHG (Figure 4i) [51,87]. This is due to the interlayer charge transfer between 2 LM and 1 LG, as well as the unbalanced charge distribution within 2 LM, resulting in the breaking of the centrosymmetric structure.

Interestingly, Yang et al. [84] discovered that artificially twisted bilayer graphene (tBLG) structures can also induce SHG (Figure 4j). The twisting angle between two monolayers in van der Waals structures provides a certain degree of freedom in controlling the optical properties of two-dimensional materials. The interlayer twist plays a crucial role in adjusting the bandgap and controlling the overall symmetry of the material. Determined by the twist angle, the magnetization tensor components of tBLG’s major chirality range from 0 to 28 × 10^4^ pm^2^/V. By directly manipulating the lattice arrangement of graphene through strain engineering, strong polarization is generated between two initially balanced sublattices (Figure 4k). The polarization disrupts the sublattice symmetry of graphene, achieving a pronounced second-order response [55].

#### 3.1.2. SHG in Transition Metal Dichalcogenides (TMDs)

The family of two-dimensional materials is gradually expanding, with transition metal dichalcogenides (TMDs) being an important category among them. TMDs are classified as MX_2_-type semiconductors, where M denotes transition metal atoms including Mo or W, and X denotes chalcogen elements such as S, Se, or Te. TMDs exhibit attractive size-dependent electrical, mechanical, optical [88], chemical [89,90], and thermal properties, making them highly promising in the fields of nanoelectronics, optoelectronic devices [88], sensors [91,92], energy storage, and conversion [93].

**Figure 5:**
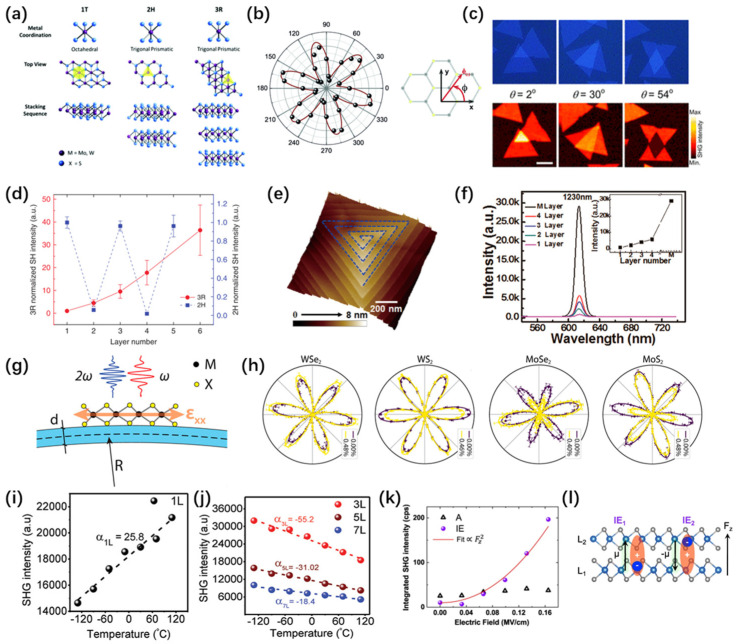
Structure of TMDs and representative studies on SHG. (**a**) The three MoS_2_ structures (1T, 2H, and 3R) exhibit distinct coordination and stacking sequences [95]. Copyright 2017, The Royal Society of Chemistry (London, UK). (**b**) A polar plot illustrating the second harmonic intensity of monolayer MoS_2_ relative to the sample angle [101]. Copyright 2013, American Physical Society. (**c**) Optical images/SHG spectra demonstrate the interlayer coupling capability of van der Waals heterostructures (twisted bilayer MoS_2_) [32]. Copyright 2014, American Chemical Society. (**d**) The dependence of SH intensity on the number of layers for 3R and 2H- MoS_2_.In the 3R crystals (red line), the SH intensity is roughly squared with the number of layers, while in the 2H crystals (blue line), the SH intensity oscillates with the number of layers [14]. Copyright 2016, CIOMP (Changchun, China). All rights reserved 2047-7538/16. (**e**) AFM image of a spiral WS_2_ nanosheet, and the blue dashed guide lines indicate the screw structures of the nanosheet. (**f**) The relationship between the SHG intensity of nanosheets and the number of layers, and the inset in (**f**) shows the parabolic increase of the SHG intensity with increasing power density. [104]. Copyright 2017, American Chemical Society. (**g**) SHG process in a strained TMD monolayer. (**h**) SHG measurements were performed on MoS_2_, MoSe_2_, WS_2_, and WSe_2_ using polarization-resolved techniques, covering both minimum and maximum applied strain levels [61]. Copyright 2018, the Authors. This article is distributed under a Creative Commons Attribution (CC BY) license. Dependence of MoSe_2_ temperature on SHG response within (**i**) 1 L and (**j**) 3 L, 5 L, and 7 L, where dashed lines indicate the linear fits; α_1_, α_3_, α_5_, and α_7_ indicate the slopes of the linear fit dashed lines for 1 L, 3 L, 5 L, and 7 L [100]. Copyright 2020, WILEY-VCH Verlag GmbH & Co. KGaA, Weinheim, Germany. (**k**) Comparison of the SHG signals of the interlayer exciton IE (solid circles) and the intralayer exciton A (open triangles) as a function of the applied electric field $F_{Z}$ The SHG amplitude of IE is fitted by the quadratic function ∝$F_{Z}^{2}$ (red line). (**l**) A diagram illustrating a bilayer with two distinct interlayer configurations [46]. Copyright 2021, the Authors.

Due to the distinct coordination layers of transition metal atoms, monolayer TMDs typically present octahedral or trigonal prism coordination phases [29,60,94,95,96]. Multilayer TMDs give rise to diverse polymorphic structures, as each layer can adopt either of the two coordination phases independently. Three common crystalline structures, labeled 1T, 2H, and 3R [97,98], are distinguished based on the number of layers in the crystal cell and the exhibited symmetry type (Figure 5a). TMDs in the 1T phase typically exhibit a metallic nature with a triangular structure. On the other hand, 2H-phase TMDs feature an AB stacking structure [3,46], where metal atoms and neighboring layers of dichalcogenide atoms are precisely arranged together, making it one of the most extensively studied systems. The metallic properties of the 1T phase and the maintained inversion symmetry in even layers of the 2H phase make these structures unfavorable for generating second harmonic generation. Conversely, TMDs in the 3R phase with ABC stacking possess a non-centrosymmetric structure, and the breaking of inversion symmetry triggers a significant effective second-order nonlinear polarization [99,100] (Table 2).

SHG is a simple, easy, and quick way to identify various characteristics of TMD materials, such as lattice symmetry and orientation [41,46,99,101,107,110], stacking angles [36,42], grain boundaries [103], and layer numbers [99,101,108]. In 2013, Malard et al. [101] first revealed the fundamental symmetry and orientation of MoS_2_ crystals using SHG (Figure 5b). Subsequently, Hsu et al. [32] established the relationship between second harmonic intensity, polarization, and stacking angles, providing a detailed characterization of grain boundaries (Figure 5c). Zhao et al. [14] investigated the layer dependency of SHG in 3R and 2H-MoS_2_. Since monolayer 2H-MoS_2_ belongs to the D_3h_ point group and exhibits inversion symmetry breaking, the inversion symmetry is restored when the number of layers increases to two in bilayer MoS_2_. Therefore, as the number of layers increases, odd-numbered layers can generate SHG responses, while even-numbered layers disappear. In the case of the 3R phase, there is a misalignment between the upper and lower layers of MoS_2_, resulting in inversion symmetry breaking even in even-numbered layers. Thus, both odd and even-numbered layers exhibit significant SHG responses (Figure 5d).

Building upon this foundation, researchers conducted in-depth investigations into the impact of stacking methods [53,58,104], strain [61], temperature [100], and electric fields [46] on SHG. In 2017, Fan et al. [104] proposed a novel helical WS_2_ structure (Figure 5e). Research has found that due to the symmetry breaking in the twisted screw structures, the SHG intensity rapidly increases with the number of layers (Figure 5f). This is completely different from conventional 2H-stacked transition metal dichalcogenides (TMDs), where the SHG intensity shows an odd–even relationship with the number of layers. In 2020, a systematic study was conducted on the robust SHG signal efficiency of helical TMDs, along with its correlation with intrinsic band characteristics [58]. Mechanical strain can reduce the symmetry of crystals, and even weak strains can have a significant impact on the SHG intensity of different polarization directions. Therefore, Mennel et al. [61] experimentally determined the second-order nonlinear optical susceptibility tensors of monolayer MoS_2_, MoSe_2_, WS_2_, and WSe_2_ under an excitation wavelength of 800 nm(Figure 5g,h). Interestingly, Khan et al. [100] discovered a temperature-dependent second harmonic generation trend that exhibited opposite behavior between single-layer and select odd-layer (3 L, 5 L, 7 L, etc.) TMDs. For instance, while 1 L MoSe_2_ displayed a substantial SHG enhancement (25.8%) with increasing temperature, some odd-numbered layers exhibited significant SHG suppression, with percentages of −55.2%, −31.02%, and −18.4% for the 3 L, 5 L, and 7 L of MoSe_2_, respectively (Figure 5i,j). Similar trends were observed in other TMD materials such as MoS_2_, WS_2_, and WSe_2_. This temperature-dependent SHG behavior can be explained by the thermal expansion effects in monolayer and multilayer TMDs. And Shree et al. [46] demonstrated robust and adjustable exciton-mediated harmonic signals in 2H MoS_2_ bilayers. This was achieved by manipulating the excitation laser energy, dielectric environment, and applied electric field, surpassing the non-resonant harmonic signals in monolayers (Figure 5k,l).

Furthermore, TMDs can form vertical or lateral heterostructures through vertical stacking or lateral stitching, which can be performed on virtually any substrate, enhancing the flexibility and operability of fabrication [111]. The van der Waals heterostructures formed between TMDs introduce new symmetries, leading to the emergence of additional nonlinear coefficient elements and thereby offering new possibilities for controlling nonlinear optical effects. Therefore, the synthesis of such heterostructures is crucial. Alloying is divided into direct and indirect methods, representing indispensable approaches for adjusting material bandgaps by utilizing the kinetics and thermodynamics of alloy reactions to synthesize lateral and vertical heterostructures of high-speed steels. Based on this, methods such as edge epitaxy, photolithographic patterning [112], and electron beam epitaxy have been derived. Hossein et al. [113] combined experimental and density functional theory (DFT) calculations to reveal the defect-mediated mechanism of alloying in single-layer TMD crystals, providing excellent theoretical guidance for synthesizing advanced alloys through defect engineering. Le et al. [16] studied the effect of Se alloying on the SHG properties in single-layer MoS_2_, finding that alloying MoS_2_ with Se can further enhance and broaden the overall SHG efficiency.

In a study on controlling SHG in TMD heterostructures, Li et al. [114] designed a one-dimensional heterostructure comprising TiO_2_ nanowires and monolayer MoS_2_. They found that the SHG intensity in the overlapping region was enhanced by approximately 10 times. Additionally, the anisotropic SHG polarization patterns in the overlapping region exhibited a dependence on the stacking angle between the nanowires and MoS_2_ crystal orientation. This implies that SHG can be effectively controlled by changing the polarization direction of the incident light or the stacking angle, achieving anisotropic enhancement of SHG. Subsequently, He et al. [115] computationally investigated the physical properties of two types of van der Waals heterostructures, MoTe_2_/WSe_2_ and MoSe_2_/WSe_2_, using first-principles calculations. Their work provides theoretical guidance for the application of van der Waals heterostructures in tunable nonlinear optoelectronic devices. Zheng et al. [116] observed deformation in the polarization pattern of SHG in MoS_2_/CrOCl heterostructures, indicating a change in the crystal symmetry of MoS_2_. This was attributed to uniaxial strain caused by lattice mismatch, leading to the breaking of rotational symmetry in MoS_2_ and consequently altering the polarization properties of SHG.

Although current research demonstrates the significant potential of TMD heterostructures in SHG, the reliability and repeatability of their synthesis techniques still need further improvement. A deeper understanding and precise control of the dynamics and thermodynamics of the synthesis are crucial for pushing TMD heterostructures toward their fundamental limits.

#### 3.1.3. SHG in Group IV Monochalcogenides

**Figure 6:**
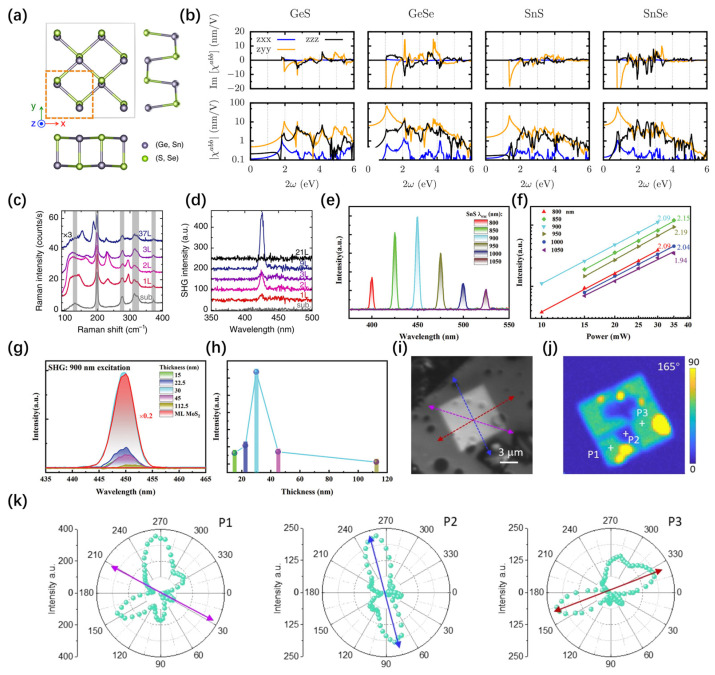
Structures of group IV monochalcogenides and representative studies on SHG. (**a**) The atomic configuration of the group IV monochalcogenide MX monolayer [129]. Copyright 2017, American Chemical Society. (**b**) SHG tensor $\chi_{2}^{abb}(-2\omega ,\omega ,\omega )$ for monolayer monochalcogenides MX = GeS, GeSe, SnS, and SnSe with the outgoing photon frequency 2*ω* [130]. Copyright 2017, IOP Publishing Ltd. (Bristol, UK) (**c**) The relationship between the number of layers and Raman spectra of SnS at 3 K. (**d**) SHG spectra for SnS at room temperature ranging from bulk to monolayer thicknesses [131]. Copyright 2020, the Authors. (**e**) SHG for the 30 nm thickness SnS film at different excitation wavelengths. (**f**) SHG power variation at excitation wavelengths from 800 to 1050 nm. (**g**) SHG intensities of SnS thin films of various thicknesses and monolayer MoS2 (ML) under 900 nm laser excitation were compared. (**h**) The variation in SHG intensity with thickness [57]. Copyright 2021, Wiley-VCH GmbH. (**i**) Low-resolution STEM image depicting the SnSe flake. The pink, blue, and red lines represent the direction of the strongest SHG intensity on the polar plots in (k) on P1, P2, and P3, respectively (**j**) SHG mapping of a SnSe flake with the incident fundamental polarization rotated by 165° relative to the horizontal axis of the image. (**k**) SHG intensity dependence on polarization rotation angle at P1, P2, and P3 [50]. Copyright, 2023 Wiley-VCH GmbH.

Currently, it has been predicted that group IV monochalcogenides, specifically GeSe, GeS, SnSe, and SnS (denoted by MX where M = Ge, Sn and X = Se, S), will display significant in-plane spontaneous electric polarization [117,118,119,120] and notable shift-current response [120,121,122]. Moreover, with abundant reserves, low toxicity, and chemical stability, these group IV monochalcogenides find widespread applications in diverse fields, including optoelectronics [123,124], thermoelectrics [121,125,126], and ion batteries [127,128]. The crystalline structure of monolayer IV-group monochalcogenides comprises two atomic layers, exhibiting a sinusoidal pattern along the x or y direction. The original configuration possesses four-fold rotational symmetry and four mirrors [129] (Figure 6a). The monolayer MX is categorized under the noncentrosymmetric point group *C_2v_* (mm2), possessing up to five distinct SHG susceptibility tensor elements. Conversely, their bulk counterparts belong to the centrosymmetric point group *D_2h_* (mmm), resulting in no SHG response [129]. Similar to TMDs, odd layers in MX can generate SHG responses, while even layers exhibit no SHG response.

Raj Panday et al. [130], utilizing density functional methods, demonstrated that group IV monochalcogenide compounds exhibit the largest reported effective SHG to date. It can achieve magnitudes of up to 10 nm/V, which is approximately ten times greater than the typical GaAs. Figure 6b presents the DFT outcomes of the imaginary and real parts of *χ*^2^ for MX = GeS, GeSe, SnS, and SnSe. An interesting characteristic is the pronounced in-plane anisotropy of χ^2^ in MX, with $|\chi_{2}^{zyy}|\;$ typically exceeding $\left|\chi_{2}^{zzz}\right|$. Higashitarumizu et al. [131] experimentally demonstrated the in-plane ferroelectricity of micrometer-scale monolayer SnS at room temperature. Surprisingly, below 15 layers, SnS exhibits robust room temperature ferroelectricity, irrespective of the odd or even number of layers. This contrasts with the conventional notion that only odd-numbered layers break centrosymmetry to exhibit ferroelectricity (Figure 6c,d). Subsequently, Zhu et al. [57] employed molecular beam epitaxy (MBE) to prepare a few-layer SnS. They found that the dependence of thickness on SHG is closely related to the coherence length. Additionally, they obtained the second-order nonlinear susceptibility of few-layer SnS. Polarization-dependent SHG research revealed typical anisotropic patterns and was employed to ascertain the crystal orientation of the SnS film (Figure 6e–h). Mao et al. [50], through atomic structure characterization, revealed that adjacent van der Waals ferroelectric layers in SnSe exhibit both parallel and antiparallel stacking, leading to an ordered arrangement of ferroelectric or antiferroelectric domains. This ordered arrangement significantly contributes to the generation of second harmonic waves, enhancing the production of second harmonic generation (SHG) through coherent enhancement effects. Remarkably, the observed SHG resulting from this coherent enhancement is 100 times more intense than that of monolayer WS_2_. Figure 6i–k illustrates the polarization angle-dependent SHG of a SnSe flake, reflecting the combination of second harmonic fields from adjacent domains and the coherent stacking structure perpendicular to the plane (Table 3).

#### 3.1.4. SHG in Group III–VI

Materials from the III–VI groups, (i.e., GaSe, GaS, GaTe, and InSe) have attracted widespread attention because of their unique structures and outstanding optical and electronic properties [132]. Both InSe and GaSe exhibit second harmonic generation (SHG) signals [133,134], making them extensively used in saturable absorption devices in ultrafast photonics [135,136,137]. Additionally, they possess two-photon-excited fluorescence (TPEF) characteristics. GaTe crystals, owing to their superior bandgap (1.7 eV at 300K) and high atomic weight, are considered ideal window materials for radiation detectors [138,139]. GaS, with its wider bandgap, holds potential for applications in photodetectors, flexible electronic devices, solar energy conversion, and nonlinear optics [140,141,142] (Table 4).

**Figure 7:**
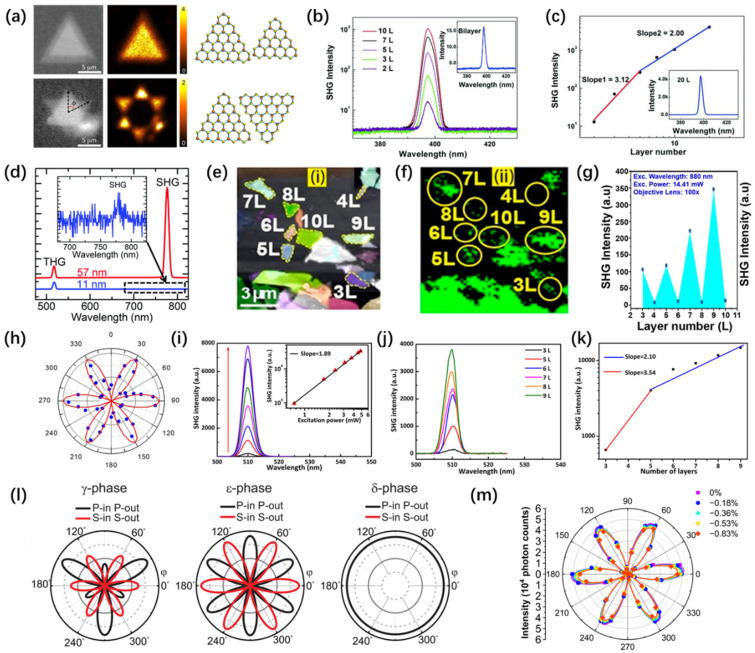
Structures of groups III–VI and representative studies on SHG. (**a**) Optical images and SHG data were obtained for AA and AB stacking configurations of β-GaSe, with Φ representing the twist angle [143]. Copyright 2015, American Chemical Society. (**b**) SHG from GaSe thin layers with variably numbered layers. (**c**) The dependency of SHG intensity on the number of layers in GaSe [144]. Copyright 2015, WILEY-VCH Verlag GmbH & Co. KGaA, Weinheim. (**d**) The SHG and THG intensities of GaTe nanosheets at different thicknesses [147]. Rights managed by AIP Publishing. (**e**) Microscopic image of the ME GaS sample with 3−10 L. (**f**) SHG mapping image of the ME GaS sample with 3–10 L thickness. (**g**) The relationship between the SHG signal and the number of layers in the ME GaS sample, with a fixed excitation wavelength of 880 nm and a pump power of 14.41 mW [146]. Copyright 2022, American Chemical Society. (**h**) SHG intensity dependency on the azimuthal angle in monolayer InSe, and the six-fold rotational symmetry suggests the three-fold symmetry in monolayer InSe [148]. Copyright 2018, IOP Publishing Ltd. (**i**) The dependence of normalized SHG intensity on laser excitation power in few-layer pure *ε*-InSe. (**j**) SHG spectra obtained from the ε-InSe nanosheet with an excitation wavelength of 1020 nm. (**k**) The dependence of SHG intensity on the number of layers in few-layer pure *ε*-InSe [133]. Copyright 2019, American Chemical Society. (**l**) Simulated SHG responses of γ-, ε-, and δ-phase InSe under oblique incidence, and the black line is P-in P-out, the red line is S-in S-out [150]. Copyright 2022, Wiley-VCH GmbH. (**m**) Angle-resolved SHG of InSe under varying levels of strain [151]. Copyright © 2023, the Authors. Licensee MDPI, Basel, Switzerland.

Researchers explored the symmetry and orientation of monolayer GaSe by examining the polarization-dependent second harmonic generation (SHG) intensity. They found that for bilayer GaSe, SHG strongly depends on the stacking mode: the AA stacking mode (ε-phase) breaks inversion symmetry, leading to SHG signals, while the AB stacking mode (β-phase) does not generate SHG signals (Figure 7a) [143]. Additionally, ε-GaSe exhibits observable frequency-doubling effects in both odd and even layers of nanosheets. The SHG response of GaSe with a thickness less than 5 L shows a nearly cubic dependence on the number of layers, while for thicknesses exceeding 5 L, it demonstrates a quadratic dependence (Figure 7b,c) [144,145].

Like *ε*-GaSe, GaTe, as a non-centrosymmetric two-dimensional layered material, can generate SHG response regardless of thickness. When excited by a 1560-nanometer femtosecond fiber laser, the SHG signal of GaTe strongly depends on the layer count (Figure 4d) [147]. In contrast, the SHG signal of *β*-GaS is constrained by the oddness of the layer count; odd layers of *β*-GaS belong to the $D_{3h}^{1}$ space group, exhibiting non-centrosymmetric properties and thus producing SHG signals. Meanwhile, layers with an even count are associated with the $D_{3d}^{3}$ centrosymmetric space group, resulting in the absence of detected SHG signals (Figure 7e–g) [146].

Because of the weak vdW interlayer coupling, InSe’s crystal structure displays polymorphism, characterized by four distinct stacking orders (*γ*-, *ε*-, *β*-, and *δ*-phases). Researchers initially investigated the dependence of second harmonic generation (SHG) intensity on the azimuthal angle and emission power for single-layer γ-InSe and ε-InSe (Figure 7h,i) [133,148,149]. Subsequently, they explored the functional correlation between SHG intensity and the layer count (Figure 7j,k) [133,149]. Sun et al. simulated the SHG signals produced by *γ*, *ε*, and *δ* phases of InSe under oblique incidence, experimentally confirming the dominance of the *ε*-phase in the InSe crystal (Figure 7l) [150]. Additionally, Li et al. employed first-principles calculations to examine how strain affects the SHG sensitivity and the matching angle-resolved SHG patterns in *γ*-InSe. Both experimental and computational findings suggest a decreasing trend in SHG intensity with an increase in uniaxial strain on the InSe monolayer (Figure 7m) [151].

### 3.2. LD Halide Perovskites

Metal halide perovskites are characterized by a chemical formula of ABX_3_, where A and B denote cations of varying sizes, and X signifies halide anions. The selection of organic and inorganic components allows for the tuning of bandgaps and the diversification of structural forms in metal halide perovskites. In addition to their three-dimensional counterparts, layered two-dimensional (2D) perovskites exhibit excellent carrier mobility and solution processability. Consequently, they are also utilized in the fabrication of thin-film transistors and light-emitting diodes [152]. LD halide perovskites have been synthesized by incorporating long-chain cations, serving as a barrier between inorganic octahedral frameworks. This approach has garnered significant interest due to its superior optical properties, making it particularly suitable for photovoltaic and optoelectronic applications [153,154,155,156]. Specifically, the pronounced quantum confinement effect and exciton effect in LD perovskites result in enhanced nonlinear optical responses compared with those observed in 3D phases [157,158,159]. In recent years, there have been reports of spontaneous electric polarization effects and SHG in bulk perovskites [17,18,160,161,162]. The improved stability of the LD perovskites span from 0D, 1D, and 2D in an ambient atmosphere, and the observation of this phenomenon is scarcely evident in the majority of conventional perovskite materials, which align with their centrosymmetric crystal space groups, extending their investigation and application such as electro-optical and frequency doubling/mixing application in the SHG nonlinear optical process [163,164,165,166,167,168,169,170,171,172,173]. 

**Figure 8:**
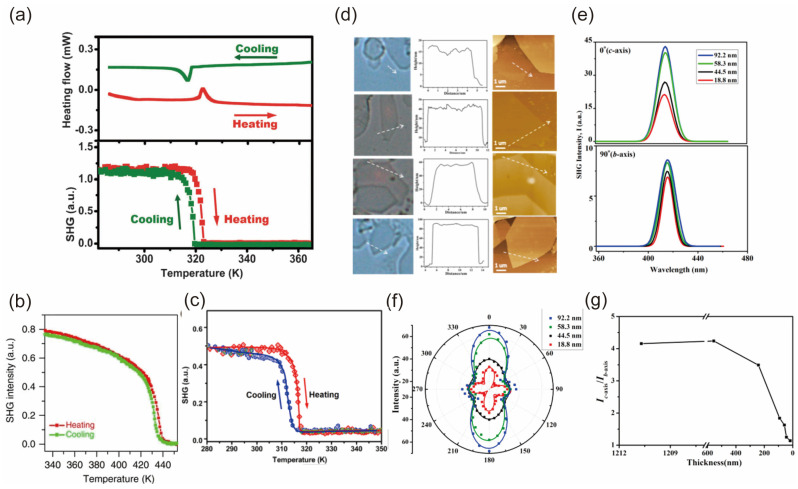
SHG variety characterization. Phase transition characterization from (**a**) 0D (N-methylpyrrolidinium)_3_Sb_2_Br_9_ [158]. Copyright © 2016 WILEY-VCH Verlag GmbH & Co. KGaA, Weinheim. (**b**) The layer (benzylammonium)_2_PbCl_4_ [168], Copyright © 2015, the Authors and (**c**) SHG intensity of (C_4_H_9_NH_3_)_2_(CH_3_NH_3_)_2_Pb_3_Br_10_ with different temperatures [174]. Copyright © 2017 Wiley-VCH Verlag GmbH & Co. KGaA, Weinheim. (**d**) From left to right images are microscopic graphs, height profiles, and topographies of measured (C_6_H_5_CH_2_NH_3_)_2_PbCl_4_ nanosheets with lateral sizes of approximately 10 µm. (**e**) The spectra of SHG signals oriented to the *c*-(0°, above) and *b*-axis (90°, below) collected from (C_6_H_5_CH_2_NH_3_)_2_PbCl_4_ nanosheets of varying thickness. (**f**) Polar SHG intensity plots of the analyzed nanosheets. (**g**) Thickness dependence of SHG intensity anisotropy (*I_c-axis_*/*I_b-axis_*) of measured nanosheets [169]. Copyright © 2019 American Chemical Society.

Given that only non-centrosymmetric materials exhibit SHG activity, they serve as a sensitive instrument for investigating the loss of inversion symmetry during a phase transition. In this case, the SHG signal is sufficiently evident to prove the symmetry breaking during the phase transition. Meanwhile, Zhihua Sun et al. demonstrated symmetry breaking from the variable-temperature SHG effects of 0D perovskite (*N*-methlpyrrolidinium)_3_Sb_2_Br_9_ (Figure 8a) [171]. Yang Hu also performed temperature-dependent SHG to analyze the phase transition [172]. Meanwhile, Lina Li et al. investigated the hybrid ferroelectric with uncommon 2D multilayered perovskite framework (C_4_H_9_NH_3_)_2_(CH_3_NH_3_)_2_Pb_3_Br_10_, which confirmed the emergence of spontaneous polarization by SHG effects in various temperatures (Figure 8b) [174]. These effects are comparable to other hybrid ferroelectrics, such as (3-ammonio-pyrrolidinium)RbBr_3_ [175], AMX_3_-type hybrid perovskites [176], and antiperovskite ferroelectric [(CH_3_)_3_NH]_3_(MnBr_3_)(MnBr_4_) [177]. Wei-Qiang Liao et al. reported the change in SHG signal from the perovskite (benzylammonium)_2_PbCl_4_ layer, showing almost overlapping curves in the heating and cooling runs (Figure 8c) [168]. Their markedly decreased SHG signal indicates the phase transition at around 85 °C from a low-temperature SHG active structure to a high-temperature structure. Moreover, Wenjuan Wei et al. observed that the in-plane SHG intensity anisotropy of orthorhombic 2D lead halide perovskite [(C_6_H_5_CH_2_NH_3_)_2_PbCl_4_] nanosheets decreased with reduced layer thickness (Figure 8d–g) [169]. The orientations of organic components at the interface play a pivotal role in determining their electrical properties, with a specific emphasis on dipolar susceptibility.

**Figure 9:**
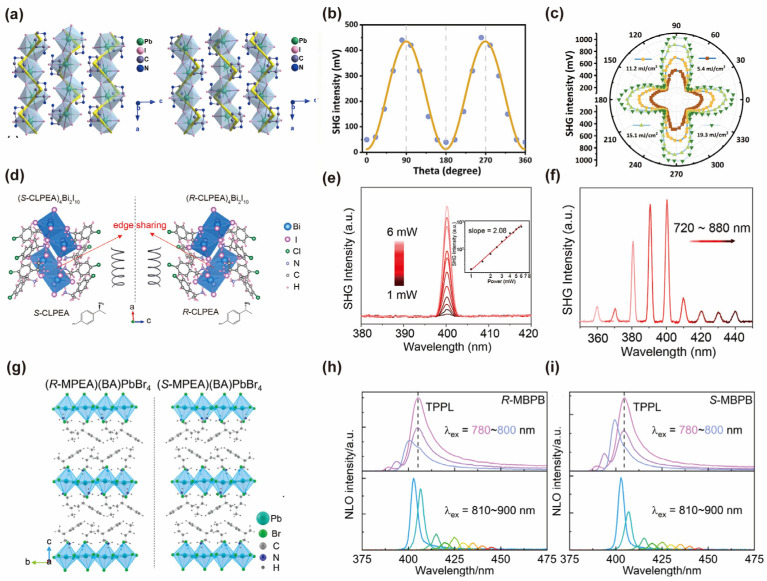
Crystal structure and second-order NLO response. (**a**) Crystal structure of [(*R*/*S*)-3-aminopiperidine]PbI_4_ [182]. (**b**,**c**) The intensity of SHG as a function of the linear polarization angle, which is tuned by employing a *λ*/2 plate. Copyright © 2021 Wiley-VCH GmbH. (**d**) Crystal structures of (*R*-/*S*-CLPEA)_4_Bi_2_I_10_ [21]. (**e**,**f**) Power-dependent and wavelength-dependent SHG intensity of the (*S*-CLPEA)_4_Bi_2_I_10_ crystal. The incident laser intensity was increased from 1 mw to 6 mw. The wavelength is changed from 720 nm to 880 nm. Copyright © 2023 American Chemical Society. (**g**) Crystal structures of chiral 2D *R*- and *S*- MBPB perovskites [179]. (**h**,**i**) SHG intensity variation in *R*- and *S*- MBPB perovskites under different excitation wavelengths from 780 to 900 nm. Copyright © 2023 American Chemical Society.

Chirality pertains to the phenomenon where an entity’s mirror image cannot be superimposed upon itself. This concept has been extensively investigated across a range of cutting-edge applications. Chiral hybrid perovskites, as a novel class of chiral semiconductors, have demonstrated significant potential in the realm of nonlinear optics, particularly in SHG [20,21,178,179,180,181]. For instance, Dongying Fu [182] and coworkers investigated the SHG-circular dichroism (SHG-CD) effect in chiral 1D [(*R*/*S*)-3-aminopiperidine]PbI_4_ bulk single crystals. The application of SHG-CD technology broadens the detection range to encompass the near-infrared region. As shown in Figure 9a–c, upon excitation by a circularly polarized laser, the SHG intensity from the crystal displays a pronounced polarization dependence. This intensity is significantly influenced by the power of the irradiation. Meanwhile, Zhao et al. designed chiral *R*-/*S*-CLPEA (CLPEA = 1-(4-chlorophenyl)ethylamine) into bismuth-based perovskites and increased the SHG and nonlinear optical circular dichroism [21]. As shown in Figure 9d–f, the positive correlation between the incident laser’s power at 800 nm and wavelength-dependent SHG effects was demonstrated. This was achieved through the second-order NLO process, which exhibited a wide response range. In recent years, Guo et al. unveiled the spatially correlated chirality and the SHG properties from *R*- and *S*-*β*-methylphenethylammonium(butylammonium)PbBr_4_ (*R*- and *S*-MBPB) 2D chiral perovskites [179]. Their efficient SHG was attributed to localized out-of-plane supramolecular orientations (Figure 9g–i). 

**Figure 10:**
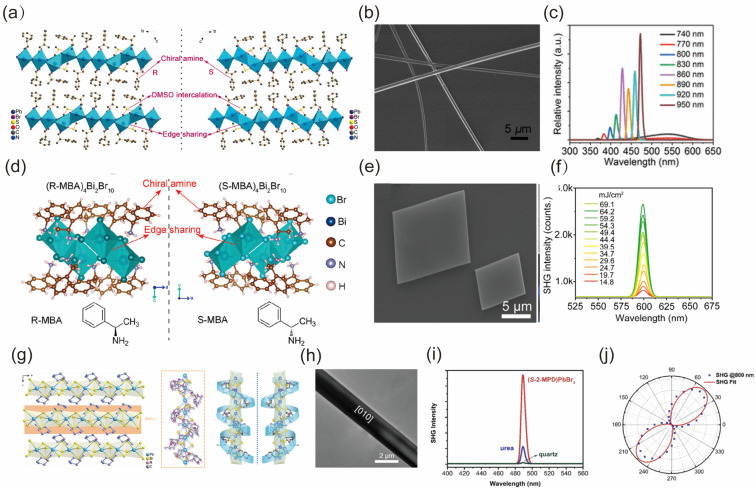
Crystallographic structure diagram and SHG properties of the nanostructured chiral hybrid perovskites. (**a**) Crystal structure of chiral perovskite, showing features of chiral, DMSO embedding, and partially shared edge [170]. (**b**) SEM image of the (*R*-MPEA)_1.5_PbBr_3.5_(DMSO)_0.5_ nanowire. (**c**) NLO spectra of a (*R*-MPEA)_1.5_PbBr_3.5_(DMSO)_0.5_ nanowire pumped under different wavelengths and normalized by the incident power. Copyright © 2018 American Chemical Society. (**d**) Crystalline structure of the grown (*R*-MBA)_4_Bi_2_Br_10_ and (*S*-MBA)_4_Bi_2_Br_10_ crystals [180]. (**e**) SEM characterization of (*R*-MBA)_4_Bi_2_Br_10_ microplates. (**f**) Power-dependent SHG spectra of the (*R*-MBA)_4_Bi_2_Br_10_ microplates (excitation wavelength: 1200 nm). Copyright © 2023 American Chemical Society. (**g**) Structure of (*S*/*R*-2-MPD)PbBr_3_ along the crystallographic *b*-axis [183]. (**h**) TEM image of a single (*S*-2-MPD)PbBr_3_ microrod crystal. (**i**) SHG intensities of (*S*-2-MPD)PbBr_3_ crystal, urea, and quartz at a wavelength of 980 nm under identical test conditions. (**j**) SHG intensity variation with polarization from a vertically oriented (*S*-2-MPD)PbBr_3_ crystal. Copyright © 2021 Wiley-VCH GmbH.

Inspired by pioneering works of functional LD chiral bulk perovskites, Xu et al. reported the construction of the chiral halide perovskite material and the observation of strong SHG responses from the nanowires of this kind of perovskite material offering a new platform for future engineering of the optoelectronics of hybrid perovskite materials [170]. The two-dimensional inorganic layer and perovskite crystal are noncentrosymmetrically assembled into a chiral P1 space group based on an organic component of chiral *β*-methylphenethylamine (MPEA), which can obtain effective SHG signals under excitation of different wavelengths (Figure 10a–c). Meanwhile, Fu et al. also synthesized the chiral hybrid bismuth halide and acquired the SHG effect from these grown chiral lead-free perovskite spiral microplates [180]. As shown in Figure 10d–f, the spiral microplates synthesized for the first time exhibit a large effective second-order NLO coefficient (*d_eff_*) up to 11.9 pm V^−1^ and a high laser-induced damage threshold of up to 59.2 mJ cm^−2^. Yongshen Zheng et al. reported superior performances in SHG of 1D chiral perovskites (*R*/*S*-2- methypiperidine)PbX_3_ (X = Cl, Br, I) microrods, including a large SHG coefficient (≈2.84 mJ cm^−2^) and high polarization ratio shown in Figure 10g–j [183].

**Figure 11:**
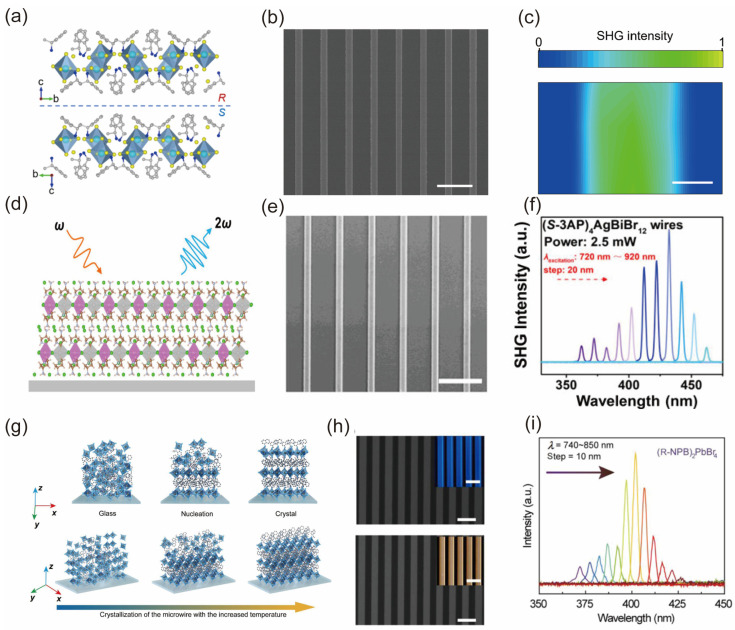
SHG properties of single-crystalline perovskite microwire arrays. (**a**) Crystal structures of the enantiomers of (*R/S*-MBA)_4_Bi_2_Cl_10_ along the a-axis [185]. (**b**) SEM image of (*R*-MBA)_4_Bi_2_Cl_10_ microwire arrays. Scale bar: 10 µm. (**c**) SHG mapping of a typical (*R*-MBA)_4_Bi_2_Cl_10_ microwire. Scale bar: 1 µm. (**d**) SHG conceptual illustration of (*S*-3AP)_4_AgBiBr_12_ microwires [186]. The red curve is incident wavelength and bule curve is the SHG signal. (**e**) SEM image of a (*S*-3AP)_4_AgBiBr_12_ single-crystalline microwire. Scale bar: 5 µm. (**f**) Wavelength-dependent SHG intensity of (S-3AP)_4_AgBiBr_12_ microwire arrays, with wavelengths ranging from 760 to 920 nm in increments of 20 nm. Copyright © 2022 American Chemical Society. (**g**) The crystal–glass phase transition process as the temperature increased [187]. (**h**) SEM image of the glassy and crystalline state of (*R*-NPB)_2_PbBr_4_ microwire arrays. Scale bar: 10 µm. (**i**) Wavelength-dependent SHG intensity of (*R*-NPB)_2_PbBr_4_ perovskite microwire arrays with excitation wavelengths varying from 740 to 850 nm. Copyright © 2022, the Authors. SmartMat published by Tianjin University and John Wiley & Sons Australia, Ltd. (Hoboken, NJ, USA).

In the development of integrated nonlinear optical devices, in addition to the low-dimensionality of materials, structural low-dimensionality, especially the array patterning, is also a key step to realize integration [170,183,184,185,186,187]. Long-range-ordered single crystal arrays, when paired with perovskite materials known for their excellent nonlinear optical properties, have unique optical anisotropy and cleavage planes that are critical to nonlinear optics, which can mitigate defects such as light scattering in the film and intrinsic dispersion refractive index of bulk materials. The increasing demand for integrated photonics circuits and chip nanophotonics has drawn heightened attention toward compact integrated devices [169,188]. Dong et al. and Yu et al. reported the synthesis of new 0D and 2D double chiral non-toxic Bi-based perovskites, (*R*/*S*-MBA)_4_Bi_2_Cl_10_ and (*R*/*S*-3AP)_4_AgBiBr_12_ featuring an observation of efficient SHG responses, attributable to the high crystallinity and pure crystallographic orientation microwire arrays. These arrays were assembled using capillary-bridge confined techniques as shown in Figure 11a–f [185,186]. Moreover, Zhao et al. have documented chiral perovskite microwire arrays that are fabricated using solution-based processes. This unique material exhibits reversible phase transitions between its glassy and crystalline states without any degradation [187]. The key advantage of this approach lies in its high SHG switching performances shown in Figure 11g–i, which enable efficient control over the generation of second harmonic signals. Moreover, these impressive results are achieved with a small footprint, making it feasible to integrate such switchable nonlinear devices into compact electronic systems.

### 3.3. Nanomaterials

In recent years, nanoparticles and nanostructured materials have attracted great interest due to their low-dimensional structures. Because of the quantum confinement, surface effects, and geometric confinement of phonons that significantly differ from those of bulk materials, they have potential applications ranging from nanoelectronics to nanophotonics. To date, SHG from nanoscale structures has been pivotal in various applications such as optical communication [189], biosensing [190], bioimaging [66,191], and laser beam control [192]. The enhancement effect of Mie resonance can be observed in several hundred nanometer nanoparticles [193]. 

**Figure 12:**
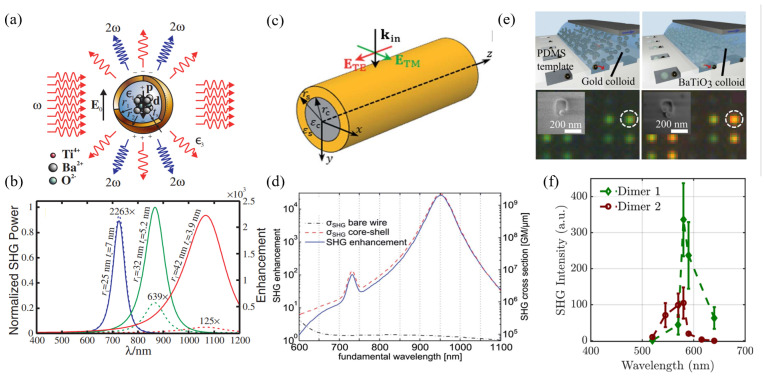
Enhancement and tunability of SHG nanostructures. (**a**,**b**) Principle and tunable plasmonic SHG of nanocavities [194]. Solid curves represent radiation power in second-harmonic frequency normalized to the maximum radiation power among the three examples. Dotted curves represent the factor of SHG enhancement compared to the core. Copyright © 2010 American Physical Society. (**c**,**d**) Considered geometry upon excitation TM polarization and the SHG enhancement of a core–shell Au-KNbO_3_ nanowire [195]. (**e**,**f**) Schematic process flow and wavelength-dependence SHG signal from BaTiO_3_-Au nanodimers [196]. The circle marks the position of a single Au nanoparticle and the same position after the BaTiO_3_ nanoparticle was added. Copyright © 2017 American Chemical Society.

To enhance SHG emission, utilizing hybrid metal–dielectric nanostructures that leverage localized surface plasmon resonance (LSPR) is an effective method [64]. The typical nanostructure shows an increased conversion efficiency to SHG called core–shell conversion, such as BaTiO_3_-Au and gold–potassium (KNbO_3_) core–shell nanoparticles (Figure 12a–d) [194,195]. However, the fabrication of such core–shell nanostructures necessitates intricate chemical synthesis techniques and precise control over the ratio of two thicknesses. Recently, Timpu et al. fabricated the hybrid metal–dielectric nanodimer, which is composed of an inorganic perovskite nanoparticle of barium titanate (BaTiO_3_) and a metallic gold (Au) nanoparticle that was shown to enhance SHG because of the LSPR of the gold nanoparticles from the BaTiO_3_ nanoparticle [196] (Figure 12e,f). They observed an average SHG enhancement of 15-fold triggered by the gold nanoparticles.

The same two-photon transitions, which can excite completely different optical properties, are involved in the new mechanism of SHG using isolated nanoparticles as the excited object [64]. Nanoparticles possessing significant photoluminescence activity demonstrate a high sensitivity to the polarization of light excitation and are not susceptible to scintillation, which is a crucial characteristic for the development of innovative microscopes [197,198,199]. Recently, nonlinear optical studies at the single nanoparticle level have been performed based on new inorganic SHG-active nanoparticles. Mugnier et al. investigated Fe(IO_3_)_3_ nanocrystal by polarization-sensitive SHG microscopy and determined the relative values of the elements to realize the orientation of individual particles in the sample obtained by optical methods [65]. This is an exceptionally appealing probe for SHG microscopy, and the orientation of each nanocrystal opens the way for a multitude of applications. As the surface-to-volume ratio escalates, surface SHG becomes increasingly dominant. In relation to nanoparticles, bulk SHG can be disregarded due to the high surface-to-volume ratio. Le and his coworkers show that a single KTiOPO_4_ (KTP) nanocrystal is an appealing material for SHG [197]. A single nano-KTP generates a fully stable, flicker-free SH signal that can be readily detected in a photon counting system under femtosecond excitation and ambient conditions. The study demonstrates that these single nanocrystals can be characterized in situ by determining their orientation relative to the optical observation axis along all three axes.

## 4. Perspective for LDM-Based SHG

### 4.1. SHG Imaging

Although TEM is one of the most powerful techniques for characterizing LDMs, it is challenging or even impossible to study thick nanostructures on nonconductive substrates. Due to the unique polarization characteristics of SHG microscopy, it can be a common technique for studying interface properties, biological imaging, and probing of noncentrosymmetric materials. The SHG-based techniques have been applied to detect semiconductor nanowires with different compositions and nanoscale morphologies. More importantly, polarization-resolved SHG microscopy has been demonstrated as an effective all-optical detection method for in situ measurements due to the polarization sensitivity of SHG to the excitation field without damaging the sample.

**Figure 13:**
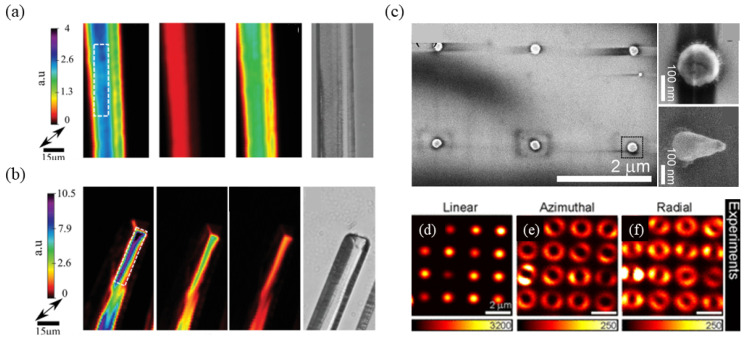
SHG images of the polarization-sensitive technique. Polarization-sensitive SHG properties (total SHG, P component, S component, and optical microscopy images) of *γ* (**a**) and *β* (**b**) glycine microneedles [200]. The black arrow indicates the incident beam polarization direction and the dashed white box outlines the integration area. Copyright © 2020 Wiley-VCH GmbH. (**c**) SEM images of gold nanobump array from the top, panel, and oblique sides. (**d**–**f**) SHG images of experiments using gold nanobumps under a focused linear pump, azimuthal pump, and radial pump (down) [201]. Copyright © 2012 American Chemical Society.

In 2020, Matthew et al. investigated the differences in SHG between susceptibility sensors of *β* and *γ* glycine microneedles. This was achieved by polarization-dependent SHG transmission microscopy [200]. As shown in Figure 13a,b, the quantitative framework presented in this study introduces a novel analytical method for the extraction of sensitivity tensor values from *β* and *γ* glycine microneedles. This analytical model is integrated with polarization-resolved SHG transmission microscopy, a technique extensively employed in quantitative SHG for material characterization and diagnostic imaging. Bautista et al. proposed an imaging method utilizing SHG accompanied by cylindrical vector beams, demonstrating exceptional sensitivity to the three-dimensional orientation and nanoscale morphology of metallic nano-objects [201]. As shown in Figure 13c–f, this technique offers contrast for structural features that are insoluble with linear methods or conventional states of polarization. It demonstrates significant potential for straightforward and cost-effective far-field optical imaging in plasmonics. 

**Figure 14:**
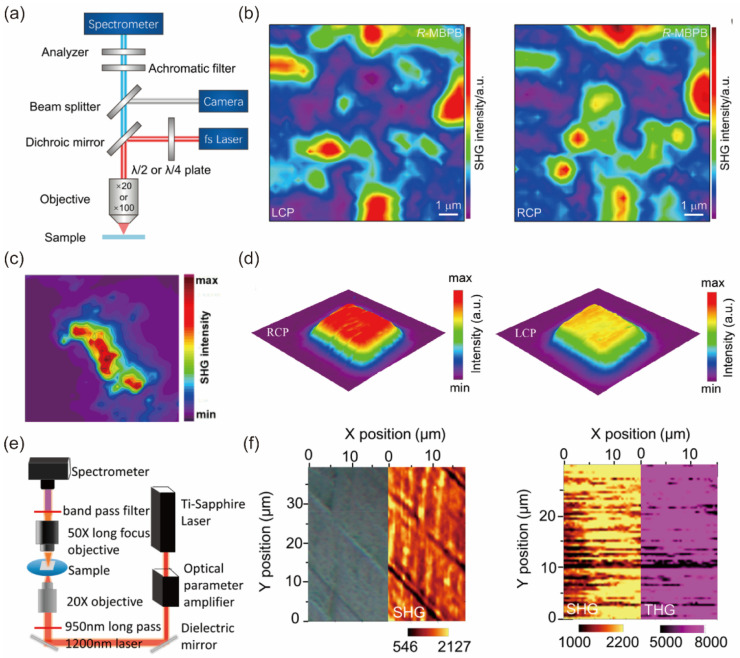
Second-order NLO mapping images of the LD perovskites. (**a**) Schematic of the SHG setup [179]. (**b**) SHG images of *R*-MBPB under LCP and RCP light excitation (10 µm × 10 µm). Copyright © 2023 American Chemical Society. (**c**) Scanned mapping image of (*R*-MPEA)_2_SnBr_6_ crystal [181]. Copyright © 2021, the Authors. Advanced Photonics Research published by Wiley-VCH GmbH. (**d**) The SHG images of the (*R*-MBA)_4_Bi_2_Br_10_ microplate at 600 nm under RCP and LCP excitation [180]. Copyright © 2023 American Chemical Society. (**e**) The second-order NLO microscopic measurement setup [20]. (**f**) The optical image, SHG mapping image, and THG mapping image (exciting wavelength is 1200 nm) of (*R*-MBA)BiI_4_ film under consistent conditions. Copyright © 2021 American Chemical Society.

As shown in Figure 14a,b, the SHG micro area images of *R*-MBPB thin films exhibited a significant position-dependent SHG intensity. Given that SHG exhibits high sensitivity to structural symmetry, this pronounced position-dependent SHG intensity is likely attributable to the polycrystalline structural characteristics. The obviously different SHG response from LCP and RCP incident lasers demonstrate *R*- and *S*-MBPB chirality properties. Similarly, Liangliang Zhao [181] and his coworkers employed a custom-built apparatus, equipped with a confocal laser scanning microscope, to investigate the SHG properties of (R-MPEA)_2_SnBr_6_ as shown in Figure 14c. The resultant mapping image from the SHG signal clearly delineates the structure of this compound, thereby indicating its SHG activity as a chiral material. The polycrystalline film’s SHG signals can be significantly affected by various factors. These include the thickness of the film, the size and orientations of the grains, and the surface characteristics of the film itself (Figure 14d) [202]. The relationship between the SHG intensity and film thickness (*L*) for chiral films, taking into account both the attenuation of the NLO signal and phase mismatch in the computation of Maxwell’s nonlinear equation, can be articulated as follows:

(6) $$I_{2}\left(L\right)\propto \left(e^{-\alpha_{2}L}-2e^{-\frac{\alpha_{2}L}{2}}\cos\left(∆k^{\left(2\right)}\right)+1\right)e^{-\alpha_{2}L}$$

where *α*_2_ represents the absorption coefficient of the *2*-fold frequency signal, while Δ*k*^(2)^ denotes the wavevector mismatch between the *2*-fold frequency wave with the pump wave. They also conducted a scan of the NLO signal from a specific thick (*R*-MBA)BiI_4_ film. Upon comparing the SHG mapping with the optical image, it was observed that the SHG intensity changes in correlation with fluctuations in the thickness of the (*R*-MBA)BiI_4_ film. The images from the SHG and THG mappings continue to exhibit synchronous intensity variations (Figure 14e,f).

**Figure 15:**
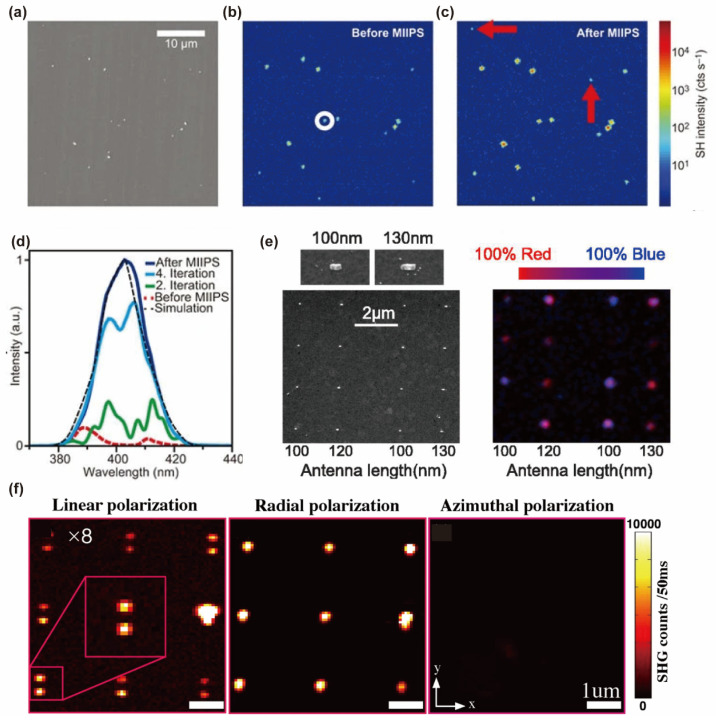
SH images on a single nanoparticle under femtosecond pulse. (**a**–**c**) SEM image, SH image, and the sample area after pulse compression image from the investigated area of a BaTiO_3_ nanoparticle sample. Red arrows indicate small NPs (with an average dimension of 125 nm) that are distinctly observable only post-pulse compression [192]. (**d**) SH spectra of the selected NP, as measured for different compensation masks during multiphoton intrapulse interference phase scan optimization. Copyright © 2014, the Authors. (**e**) SEM images and two-color SH image of an array of gold NPs of different lengths along with higher resolution SEM images of the individual 100 nm and 130 nm RPNPs [203]. Rights managed by AIP Publishing. (**f**) Experimental far-field SHG images from an array of vertical NRs (positioned at 0° orientation relative to the substrate normal) using a tightly focused incident beam employing linear *y*-polarized (*y*-LP), radially polarized, azimuthally polarized, and corresponding experimental conditions [204]. Copyright © 2016, the Authors.

The SHG exhibits sensitivity to the spectral phase of a laser pulse. It has been extensively utilized for femtosecond pulse characterization. Furthermore, the SH has found applications in both pulse characterization and compression through the implementation of the multiphoton intrapulse interference phase scan (MIIPS) technique. Acanto et al. utilized the nanometer source for SHG, which acquired phase information derived from a signal originating from an ultra-narrow nanometric volume, produced by single nonlinear nanoparticles (NPs), as opposed to bulk crystals. The conditions for SH phase matching of the NPs do not apply because of the smaller size compared with the excitation wavelength [197]. Thus, efficient SHG can be obtained over a wide wavelength range without any specific tuning size of the NPs or change in the orientation with respect to incident light. The total SH signal is increased by more than an order of magnitude for SH from a single BaTiO_3_ NP excited by a compression pulse [192]. Fluorescence microscopy, a prevalent technique in biological imaging, is adept at probing subcellular components and dynamic processes. When nanoparticles undergo two-photon excitation, they generate SH radiation. This process not only expedites the identification of suitable fluorescent markers or probe recognition but also enhances convenience (Figure 15d). To date, multicolor imaging has predominantly been confined to the utilization of fluorescent markers and remains unexplored in the context of SH NPs. The study further illustrated that resonant plasmonic nanoparticles (RPNPs) can generate distinct colors within the SH spectrum, even when utilizing an identical excitation laser. This capability could potentially facilitate multicolor SH imaging and exploit a unique opportunity to differentiate between two different RPNPs based on their SH spectra (Figure 15e) [203]. Furthermore, Abdallah et al. [204] performed a comprehensive examination of individual nanorods using a nonlinear optical microscope based on SHG. The excitation of these nanorods, with their diverse orientations, was facilitated by a tightly focused laser beam that was either linearly or radially polarized. This investigation unveiled a pronounced sensitivity of the SHG response to the orientation of these nanorods for these polarizations. As shown in Figure 15f, the periodicity of the SHG intensity distribution as a function of polarization confirms the importance of vector beams for enhancing the SHG signal and elucidating the image pattern recorded by nanostructures. This suggests that the orientation of anisotropic nanomaterials can be reliably determined using different polarization states of the incident beam in an SHG microscope.

The chiral metamaterial reflector is based on the integration of a nonlinear material with a designed plasma structure that strongly absorbs a circularly polarized wave of a spin state and has two key features, namely, chiral selective absorption and polarization preservation after reflection. And reflect circularly polarized waves of opposite spin in a way that preserves circular polarization [205]. Chiral resonance enhances the light–matter interaction under circular polarization excitation, greatly improving the ability of metamaterials to generate chiral selective signals and optical imaging in the nonlinear range. The addition of second harmonics is used to enhance the contrast of nonlinear images. Similarly, with the enhancement of the second harmonic on the metal Au surface, the incident circularly polarized light passes through to obtain a residual right-polarized light image without a geometric phase and a left-circularly polarized beam image with a geometric phase [206].

### 4.2. Time-Resolved SHG

Although previous studies of time-resolved optically pumped probes have investigated carrier relaxation under low optical excitation conditions, the electron dynamics of low-dimensional systems have not been studied in states related to amplitude, temperature, or electron excitation conditions, and the structural dynamics need further study. Mannebach et al. present the first measurements of the time-dependent structural and electronic response of 2D MoS_2_ using SHG [207]. It can be associated with the extreme electronic temperatures induced without modification of the unit cell structure from the large amplitude increases in SHG occurring on a few picoseconds time scales. Moreover, Taghinejad et al. reported the ultrafast modulation of second-order optical nonlinearities in a monolayer TMD film via the optical tuning of the photocarrier density through a set of transient linear and nonlinear characterizations [208]. The production of photoelectrons greatly reduces the possibility of interband electron transitions near highly symmetric K/K′ points in momentum space, reduces the efficiency of nonlinear frequency doubling when light interacts with excited monolayer crystals, and optically tunes the intensity of SHG signals emitted from TMDs.

Time-resolved second harmonic generation (SHG) is also a powerful tool to provide information on the process of electron/hole transfer from the interface to the conduction band. Tisdale et al. investigated the effect of thermal electron transport properties based on the size, shape, and material of the nanocrystals and the influence of the band structure on the surface of nanocrystals on the transport of hot electrons using time-resolved SHG [209]. Additionally, breaking the inversion symmetry through hot-electron dynamics can be leveraged to address the critical need for all-optical control of SHG in nanophotonics [210]. Furthermore, Wang et al. revealed a dominant role of the dark excitons to enhance SHG [205]. The amplitude and sign of the SHG modulation can be adjusted over a broad spectral range with different carrier dynamics.

Heterostructures of LDMs have unveiled intriguing properties, thereby stimulating both fundamental and applied research within the realms of optoelectronics and valleytronics applications. The influence of stacking on ultrafast charge transfer upon photoexcitation and interlayer recombination should be substantial. Accordingly, a distinct difference in exciton recombination has been noted between coherent and random stacking MoS_2_/WS_2_ heterostructures [211]. However, comprehensive measurements of the MoS_2_/WSe_2_ heterostructures reveal a significant variation in charge recombination lifetime across samples. Nevertheless, no discernible correlation with torsion angle has been observed [212]. Because of the ultrafast charge transfer within a few hundred femtoseconds even tens of femtoseconds, a novel experimental approach for examining charge transfer at the interface of LDM heterostructures interface has been proposed. This method involves the use of time-resolved optical SHG. In the prevalent linear pump–probe configuration, the transient response measured is a composite of both monolayer and heterostructure contributions. Moreover, the occurrence of charge transfer can be bidirectional simultaneously, contingent upon the excitation energy. Therefore, the tunable energy of the photon from the pump pulse and the polarization of the probe pulse allow resonant excitation of one of two materials and optionally detect the charge transfer in alternate directions [47]. By integrating polarization and time-resolved measurements, we can perform highly accurate and systematic analyses. This approach allows us to correlate the observed transient changes in the SH response with the inherent structure.

## 5. Summary and Outlook

In conclusion, SHG serves as a robust, versatile, and straightforward method for unveiling the physical characteristics of low-dimensional materials. Leveraging its profound sensitivity to both spatial inversion and time-reversal symmetry, herein we systematically reviewed SHG properties and applications in three kinds of low-dimensional materials, i.e., 2D materials, low-dimensional perovskite, and nanomaterials. We also elaborated the research process on SHG microscopy and time-resolved SHG of LDMs, focusing on two key aspects: symmetry breaking and enhancement of light–matter interaction. Nevertheless, the accurate identification of homo- and heterostructures in LDM systems remains a formidable challenge, particularly when considering systems with fully controlled symmetry breaking at arbitrary angles over expansive areas. Research into NLO properties based on LDMs remains in its early stages, presenting both potential opportunities and challenges. Owing to their robust quantum confinement and excitonic effects, LDMs have become a central focus of nonlinear media research. It is anticipated that with increased investment and effort, further advancements will be realized. Especially, in the context of 2D materials and layered halide perovskites, it is observed that by employing defect engineering and using organic ligands as spacer molecules, the assembly of metal halide octahedra can be alternately controlled. This control over the assembly process allows for the attainment of robust second-order NLO properties, which can be fine-tuned through structural modifications.

Nevertheless, many challenges of LD SHG materials still exist. First, the material synthesis process for SHG enhancement is still a challenge. Advances in nanofabrication have offered a new class of composite media, creating core–shell nanostructures and metamaterials whose SHG properties are determined by the shape and arrangement of their component [213,214]. And the origins of second harmonics warrant comprehensive analysis. While the well-established inherent lattice asymmetry is a primary contributor to second harmonic generation, it is important to note that surface/grain boundary and thermal lattice fluctuations can also disrupt symmetry, leading to pronounced second harmonic effects. Furthermore, the relative spatial and temporal inversion symmetries can be manipulated by controlling the electric and magnetic fields. This manipulation allows for the modulation or even enhancement of the SHG response of the LDMs. Additionally, the SHG signal can be activated and deactivated by a gate. This feature significantly broadens the versatility of contemporary photonic systems. Moreover, precisely regulated resonant wavelength limits two aspects, which are the resonance coupling SHG response based on excitons and the exciton–polariton resonance effect of LDMs, respectively. This limitation consequently restricts their practical applications. Despite significant advancements in the modulation and enhancement of SHG for LDMs, ample opportunities remain for further exploration. The investigation into the formation of magnetic ordering, magnetic domains, and ultrafast magnetic dynamics using SHG remains in its nascent stage.

Secondly, the detection of transient hole transport in perovskites has been achieved for the first time. This was accomplished through the direct detection of transient electric field migration using time-resolved micro-optical second harmonic generation (TRM-SHG) [161]. The TRM-SHG technique was utilized to examine the impact of traps on transient carrier motion. By analyzing the peak of the transient electric field distribution, we were able to separately estimate trap density and dynamic carrier mobility. Alexandra reported a time-resolved, phase-sensitive second harmonic generation (SHG) method to investigate the excited state dynamics of interfacial species [215]. The primary characteristics of this technique include its superior phase stability and sensitivity, coupled with relatively brief data acquisition periods. Optical SHG is also a reliable, non-destructive, and contactless technique for probing charge densities of the semiconductor/dielectric interface [216]. This optical method provides a new method for simple and effective measurement that can be used to characterize semiconductor interfaces in detail and to simulate experimental data using numerical solvers to extract the electronic properties of semiconductor interfaces. The method offers several benefits, including its sensitivity to crystal symmetry, non-contact nature, non-invasive approach, lack of fabrication requirements, and straightforward operation.

Thirdly, while this review primarily concentrates on the experimental process and application aspects of SHG research in LDMs, it is also beneficial for researchers to explore the theoretical modeling of SHG in LDMs. Furthermore, the emergence of 2D perovskite heterostructures provides a novel avenue for enhancing performance. The theoretical support of SHG of LD perovskite heterojunction is more significant to establishing a tunable barrier for charge carrier transport and the optimization of the photogate effect to improve performance. Comprehensively including the significant substrate effect in the modeling of SHG in LDMs is also a problem that needs to be solved. The significance of interfaces and substrates naturally amplifies atomic thin crystals. In the future, it is imperative to appropriately model and explore photon boundary conditions, mechanical strain, charge transfer, and other interface factors.

In conclusion, the future prospects for second harmonic generation in low-dimensional materials appear to be promising. This holds significant potential for advancing SHG techniques and corresponding subsequent applications, thereby compelling us to invest substantial resources toward both experimental research and practical implementation.

## Figures and Tables

**Table 1:** SHG properties of graphene.

Material	SHG Phenomena	$\chi^{\left(2\right)}$ $\left(10^{-12}m/V\right)$	Emission Wavelength (nm)	Material Characteristics		Substrate	Ref.
				Fabrication Method	Thickness of Sample Investigated		
Graphene	Current induced	120	3100	-	1 L	-	[80]
	Current induced	200	370	Thermal annealing	4 L	SiC	[81]
	Doping	22	653	CVD	1 L	Fused Silica	[79]
	Doping	-	-	CVD	2 L	SiO_2_/Si	[82]
	Stacking induced	90	650	Exfoliation	3 L (ABA)	SiO_2_/Si	[83]
	Stacking induced	-	532	Exfoliation	4 L (ABAB)	SiO_2_/Si	[54]
	Twisting	424	532	Exfoliation	2 L	SiO_2_/Si	[84]
	non-uniformly straining	-	517.5	Exfoliation	1 L	Al_2_O_3_	[55]

**Table 2:** SHG properties of TMDs.

Material	$\chi^{\left(2\right)}\left(10^{-12}m/V\right)$	Emission Wavelength (nm)	Material Characteristics		Substrate	Ref.
			Fabrication Method	Thickness of Sample Investigated		
MoS_2_	120	435	Exfoliation	1 L	Quartz	[101]
	29.5	440	Exfoliation	3 L	Quartz	[101]
	~105	405	Exfoliation	1 L	SiO_2_/Si	[34]
	~5000	405	CVD	1 L	SiO_2_/Si	[34]
	430	580	CVD	1 L	SiO_2_/Si	[102]
	2	780	CVD	1 L	SiO_2_/Si	[103]
WS_2_	4500	415	Exfoliation	1 L	SiO_2_/Si	[41]
	500	440	CVD	1 L	SiO_2_/Si	[104]
	460	532	Exfoliation	1 L	Quartz	[105]
MoSe_2_	50	810	CVD	1 L	SiO_2_/Si	[106]
	7800	775	Exfoliation	1 L	Si waveguide	[107]
WSe_2_	100	775	Exfoliation	1 L	SiO_2_/Si	[108]
	19	775	Exfoliation	5 L	SiO_2_/Si	[108]
	1000	~443	Exfoliation	1 L	SiO_2_/Si	[59]
MoTe_2_	2500	775	Exfoliation	1 L	SiO_2_/Si	[109]

**Table 3:** SHG properties of group IV monochalcogenides.

Material	$\chi^{\left(2\right)}\left(10^{-12}m/V\right)$ (Emission Wavelength)		Material Characteristics		Substrate	Ref.
	Experimental	Simulation	Fabrication Method	Thickness of Sample Investigated		
GeSe		7368 (939 nm)	-	1 L	-	[129]
		500–10,000 (620–1550 nm)	-	1 L	-	[130]
GeS		200–8000 (443–1550 nm)	-	1 L	-	[130]
SnSe		200–10,000 (620–1550 nm)	-	1 L	-	[130]
SnS		550–7800 (550–1550 nm)	-	1 L	-	[130]
	1.37 (450 nm)		MBE	~30 nm	MgO	[57]

**Table 4:** SHG properties of groups III–VI.

Material	$\chi^{\left(2\right)}\left(10^{-12}m/V\right)$	Emission Wavelength (nm)	Material Characteristics		Substrate	Ref.
			Fabrication Method	Thickness of Sample Investigated		
GaSe	2400	605	CVD	1 L	Fused silica	[143]
	1700	675	CVD	1 L	Fused silica	[143]
	700	800	CVD	1 L	Fused silica	[143]
	30	400	Exfoliation	2 L	SiO_2_/Si	[144]
	18	780	Exfoliation	Bulk	Si	[145]
GaS	47.98	440	Exfoliation	3 L	Quartz	[146]
GaTe	1.15	780	Exfoliation	14 nm	SiO_2_/Si	[147]
InSe	639	400	PVD	1 L	SiO_2_/Si	[148]
	13	400	Exfoliation	Bulk	SiO_2_/Si	[133]
